# An in-depth introduction to arts-based spiritual healthcare: Creatively seeking and expressing purpose and meaning

**DOI:** 10.3389/fpsyg.2023.1132584

**Published:** 2023-03-17

**Authors:** Carlos Laranjeira, Ana Querido

**Affiliations:** ^1^Department of Nursing Science, School of Health Sciences of Polytechnic of Leiria, Leiria, Portugal; ^2^Centre for Innovative Care and Health Technology (ciTechCare), Polytechnic of Leiria, Leiria, Portugal; ^3^Comprehensive Health Research Centre (CHRC), University of Évora, Évora, Portugal; ^4^Center for Health Technology and Services Research (CINTESIS), NursID, University of Porto, Porto, Portugal

**Keywords:** art-therapy, positive psychology, spiritual care, healthcare professionals, meaning

## Introduction

Art therapy entails a therapeutic relationship that allows the expression and exploration of mental and spiritual needs through art (Bell, 2011). It integrates artistic creation, making use of different artistic mediators (Martins, 2013). Through various forms of artistic expression, a person communicates and explores their feelings and intimacy (Lev, 2020). Creative development allows the individual to break forms of resistance, recognize their own capabilities, and feel greater self-esteem and wellbeing (Hu et al., 2021; Ryff, 2021). Exercising creativity enables a differentiated view of oneself and favors the encounter of alternative points of view regarding psychological and spiritual issues (Alvarenga et al., 2018). The aim is personal growth and contact with our sensitive world and emotional development. In the art-therapeutic process, the artistic experience through creation enriches the imaginary and contributes to self-knowledge, driving the elaboration of internal contents and personal transformation (Martins, 2013; Ettun et al., 2014). In the art-therapeutic setting, different artistic mediators facilitate creation, expression and communication, including plastic, musical, dramatic, body, literary, and playful expression. These constitute the technical and material basis for creation. Considering the particular symbolic, expressive, and creative characteristics of different resources and understanding the therapeutic potential of each resource and how they can meet individual needs is fundamental to effective Art Therapy.

Art therapy can contribute to the knowledge, recognition and appropriation of one's own spirituality. This is a necessary part of the discovery process for those on the path of transcendence, leading the individual to (re)discover a broader meaning for existence (Sánchez García and Pinna-Perez, 2021). Understanding the meaning of life is the essential and personal quest of every individual; it is a required search that leads to the path of self-knowledge.

This opinion article presents an in-depth discussion of the potential of arts-based spiritual care, realizing in advance that there is a lack of conceptual clarity. Knowing how we might embrace a conception of spirituality anchored in art therapy may allow for more holistic practices, and thereby integrate them into a larger approach to spiritual care provision.

## The creative art therapies

Art therapy is a therapeutic treatment for personal development, which uses and integrates different artistic mediators, such as paint, clay, stone, cloth and yarn, poetry or writing, music, dance and movement, storytelling, and drama [Ettun et al., 2014; British Association of Art Therapists (BAAT), 2015; Chiang et al., 2019]. All these means of artistic creation can reach the human spirit during their psycho-spiritual search for wholeness (Niemiec et al., 2020). An overview of spiritually-oriented art therapy interventions can help understand how they make transformation and healing possible by integrating the spiritual aspect into therapy (Kirca, 2019).

The therapeutic relationship is established through interaction between the client—the “creator” of the art object, which is the “creation” performed in the setting—and the observer (art therapist), using resources such as imagination, symbolism, and metaphors. This context facilitates communication, reorganization of internal contents, meaningful emotional expression, and deepening of internal knowledge, freeing the ability to think and creativity (de Witte et al., 2021). Therefore, Art Therapy presupposes a “relationship,” whose dynamic is triangular: between the patient, the creation, and the therapist. The act of creation does not happen by itself, but through the establishment of a bond of trust provided by the therapeutic alliance, in a dialogic relationship (Gazit et al., 2021).

Despite focusing on communication through artistic forms of expression, art therapy is sometimes incorrectly labeled as a “non-verbal” therapy. This misunderstanding occurs because verbal communication is not essential to the elaboration of artistically expressed content or fundamental in art therapy. Music, body movement, or playful games can become the main form of communication in therapy, transmitting deep feelings without words. Art Therapy can be enjoyed solely as non-verbal expression, for instance, by children and adolescents with psychosocial problems (Bosgraaf et al., 2020); by the elderly person who has lost the ability to speak because of a stroke or dementia (Deshmukh et al., 2018); or even a victim of trauma (Kaimal et al., 2021), who may be unable to put her ideas verbally. However, according to Malchiodi (2020), art therapy includes both non-verbal and verbal communication. In most situations, the process involves verbal expression of thoughts and feelings to help individuals make sense of their experiences, feelings, and perceptions.

Art therapy thus results in a creative process, which can also be a therapeutic process, i.e., “art as therapy.” The experience of making art offers an opportunity for imaginative expression, in an authentic and spontaneous way. A process that, over time, leads to personal fulfillment, emotional repair, and transformation at a psychic level. A holistic and extensive view of the human being is employed in Art Therapy, a therapeutic practice that aims to rescue not only the integral dimension of the subject but also their processes of self-knowledge, transcendence, and personal transformation (Gerber et al., 2018). The aim is also to promote image production, creative autonomy, the development of communication, the appreciation of subjectivity, freedom of expression, the reconciliation of emotional problems and their cathartic function (Gabriel, 2021). Notably, art therapy may be a cost-effective form of psychological therapy when compared to more traditional talking therapies (Uttley et al., 2015; Braito et al., 2022).

Art therapy is applied to people of all ages, with different psychological and medical problems. Interventions are carried out in groups or individually, in a private setting or in different institutions, such as schools, hospitals, shelters, nursing homes, day centers, etc. Art therapy stands out for its transdisciplinarity and its wide application, with little or no contraindications and, therefore, the intervention must be planned in order to specifically meet the needs of the population in question. Indeed, art therapy should not be considered as an alternative to the conventional care and treatment provided by healthcare care teams, but rather as a complementary part of a treatment plan, given its potential to improve holistic care.

## Art, healing, and care for the spirit

Theoretical perspectives emphasize spirituality and transcendence as vital components of human personality, self-awareness, developmental tasks, and wellbeing (Kruse and Schmitt, 2019). Spirituality is widely characterized as how people seek and express meaning and purpose, how they feel connected to the present, to themselves, to others, to nature, and to the meaningful or sacred (Puchalski et al., 2014). Spiritual Care should be an intrinsic element of healthcare, since spirituality is often a basic human dimension, especially during distress (Roze des Ordons et al., 2018; Fitch and Bartlett, 2019). However, implementing Spiritual Care is difficult, for it depends on the individual spirituality of the healthcare professionals and patient (or relative), and there are no quick fixes for spiritual needs, but require personal connectedness and investment (Hvidt et al., 2020).

Transcending one's boundaries is a dynamic process, which can occur in many motions, directions, and dimensions. Self-boundaries can be crossed temporally (by integrating one's past and future in a way that makes sense in the present), intrapersonally (toward self-acceptance and meaning), interpersonally (by connecting with others and one's environment), and transpersonally (by linking with dimensions beyond the typically discernible world; Reed, 2009; Post et al., 2020).

Meaning-making is encouraged when spirituality is completely accepted. Spiritual care enables the possibility and capacity to transcend the physical and psychological (Bell, 2011; Lalani, 2020), connecting people in suffering to areas of their life, frequently through imaginative artistic expression, where spirituality and religion contribute new views to human affairs.

The intersubjective and interpersonal dynamics of care offer numerous and diverse opportunities to think about and deal with spirituality. Compassionate attention to the inner world, which is endowed with transcendent and transforming aspects, is vital (Bell, 2011). This humanizing process invites thought and conversation on the intangible, abstract components of suffering. The subtleties of meaning-making, purposefulness, and affirmation of a desirable and meaningful existence all lead to and imply spirituality (Bell, 2011). The healthcare practitioner must be prepared to interpret and communicate these requirements, as well as seek appropriate aid and support to offer spiritual care (López-Tarrida et al., 2021).

Spirituality can be acknowledged, understood, and faced through art therapy, in the context of palliative, end-of-life care (Lefèvre et al., 2016; Warth et al., 2016), and other domains of mental health care (Deshmukh et al., 2018; Haeyen, 2019). The use of art materials to make drawings and paintings is a concrete, material record of the search for meaning and spiritual reflection and insight (Bell, 2011). Through the use of art materials and imagery, art therapy fosters meaning-making and so creates an intra-psychic space in which spirituality may be acknowledged, explored, and understood.

Art does not just have an accessory role but is a way of healing, which underlies the stimulation of creativity to achieve higher levels of wellbeing and the resolution of internal conflicts. Artistic creation calls upon mental functions fundamental for personal balance. The creative process' divergent thinking integrates symbolization, abstraction and a specific learning cognition that includes the sensorial and emotional system. It is a counterpoint to logical thinking. But experiences, when integrated, favor an expanded development that is fundamentally more adapted to an individual's reality. Having a more creative attitude toward life, one can respond more precisely to one's needs.

Creativity is essential for a balanced life and contributes to human flourishing. It is fundamental for finding solutions and for a subject's constructive integration into reality. The creative process cooperates by enlightening and repairing psychic processes. Creativity is defined as an existential process affiliated with the human capacity for self-realization, affirmation, and the potential to expand, develop and mature (Gosetti-Ferencei, 2020). There is no difference between creative processes related to arts and inventions and processes that develop an individual's personality, such as psychotherapy. Thus, to make the most of the capacity for creation and transformation, this capability is understood as inherent to the human being. In Art Therapy, there is an analogy between creating artistically and creating perspectives for personal change. Therefore, when creating new forms, configuring elements, and finding meanings for creation, a new ordering of life strategies is symbolically established.

The creative act imprints the mark of the creator, their originality and individuality, restoring their sense of identity, dignity, and community. Cultural and social influences are also part of this process. However, depression, fear (caused by more rigid personality traits), inner emptiness and anxiety are some of the constraints that prevent individuals, children and adults of all ages from expressing themselves creatively (Cocco, 2017; Xu et al., 2021). Creative unlocking and facilitation strategies must be provided for the development of creative activity, by connecting on an emotional level (through an empathetic relationship) or an environmental level (by finding a welcoming, comfortable space, which provides a moment of disconnection from outside influences).

Spirituality provides meaning to our lives by enhancing our consciousness of the most personal elements of our existence, material body, environment, and the divine (Bell, 2011). Creating art is a spiritual activity, or at least implies a spiritual component to existence that deserves attention and contemplation (Kirca, 2019). Creativity, imagination, and the creation of artifacts are indicators of spirituality and transcendent potential (Fotaki et al., 2020). Any psychotherapy intervention is about reinforcing elements that repair and heal the human body, mind, and spirit. Therefore, meaning-making and spirituality are essential to the realm of Art Therapy theory and practice (Bell, 2011).

## Final remarks

The meaning-making process and the self-affirmation of personal and societal values and ideas can help us understand spirituality. This domain of human experience can be explored through artistic representation, enabling people to direct their interest to the spiritual side of the creative experience. An integrative person-centered approach is a foundation for giving spirituality the same care and attention as other physical and psychological realities. It is acknowledged to have a large and decisive influence on recovery, healing, growth, and positive therapeutic outcomes. Art-based spiritual care, therefore, lends legitimacy to the care of the most vulnerable people and contributes to a caring culture focused on spiritual development. Additional research in a broader set of cultures and populations is needed to better understand the effectiveness of art therapy interventions in spiritual growth. Furthermore, conceptual clarity and consensus on terminology will be important in art-based spiritual care both in clinical and community settings.

## Author contributions

All authors listed have made a substantial, direct, and intellectual contribution to the work and approved it for publication.

## References

[B1] Alvarenga W. A. Leite A. Oliveira M. S. Nascimento L. C. Silva-Rodrigues F. M. Nunes M. D. R.. (2018). The effect of music on the spirituality of patients: a systematic review. J. Holist. Nurs. 36, 192–204. 10.1177/0898010117710855 28589782

[B2] Bell S. (2011). Art therapy and spirituality. J. Study Spirit. 1, 215–230. 10.1558/jss.v1i2.215

[B3] Bosgraaf L. Spreen M. Pattiselanno K. van Hooren S. (2020). Art therapy for psychosocial problems in children and adolescents: a systematic narrative review on art therapeutic means and forms of expression, therapist behavior, and supposed mechanisms of change. Front. Psychol. 11:584685. 10.3389/fpsyg.2020.584685 33132993 PMC7578380

[B4] Braito I. Rudd T. Buyuktaskin D. Ahmed M. Glancy C. Mulligan A. (2022). Review: systematic review of effectiveness of art psychotherapy in children with mental health disorders. Ir. J. Med. Sci. 191, 1369–1383. 10.1007/s11845-021-02688-y 34231158 PMC9135848

[B5] British Association of Art Therapists (BAAT) (2015). What Is Art Therapy? Available online at: https://www.baat.org/About-Art-Therapy (accessed December 20, 2022).

[B6] Chiang M. Reid-Varley W. B. Fan X. (2019). Creative art therapy for mental illness. Psychiatry Res. 275, 129–136. 10.1016/j.psychres.2019.03.025 30901671

[B7] Cocco G. (2017). Imagination for creative adaptation a bridge between a child's interior and Exterior world. Proceedings 1:948. 10.3390/proceedings1090948

[B8] de Witte M. Orkibi H. Zarate R. Karkou V. Sajnani N. Malhotra B.. (2021). From therapeutic factors to mechanisms of change in the creative arts therapies: a scoping review. Front. Psychol. 12:678397. 10.3389/fpsyg.2021.678397 34366998 PMC8336579

[B9] Deshmukh S. R. Holmes J. Cardno A. (2018). Art therapy for people with dementia. Cochrane Database Syst. Rev. 9:D11073. 10.1002/14651858.CD011073.pub2 30215847 PMC6513479

[B10] Ettun R. Schultz M. Bar-Sela G. (2014). Transforming pain into beauty: on art, healing, and care for the spirit. Evid. Based Complem. Altern. Med. 2014:789852. 10.1155/2014/789852 24834099 PMC4009230

[B11] Fitch M. I. Bartlett R. (2019). Patient perspectives about spirituality and spiritual care. Asia Pac. J. Oncol. Nurs. 6, 111–121. 10.4103/apjon.apjon_62_18 30931354 PMC6371668

[B12] Fotaki M. Altman Y. Koning J. (2020). Spirituality, symbolism and storytelling in twenty first-century organizations: understanding and addressing the crisis of imagination. Organ. Stud. 41, 7–30. 10.1177/0170840619875782

[B13] Gabriel R. (2021). Affect, belief, and the arts. Front. Psychol. 12:757234. 10.3389/fpsyg.2021.757234 34925160 PMC8674731

[B14] Gazit I. Snir S. Regev D. Bat Or M. (2021). Relationships between the therapeutic alliance and reactions to artistic experience with art materials in an art therapy simulation. Front. Psychol. 12:560957. 10.3389/fpsyg.2021.560957 34335345 PMC8316854

[B15] Gerber N. Bryl K. Potvin N. Blank C. A. (2018). Arts-based research approaches to studying mechanisms of change in the creative arts therapies. Front. Psychol. 9:2076. 10.3389/fpsyg.2018.02076 30443230 PMC6223244

[B16] Gosetti-Ferencei J. A. (2020). “Life as a work of art: the existential need for creativity,” in Being and Becoming: An Existentialist Approach To Life, Guides to the Good Life Series, ed J. A. Gosetti-Ferencei (New York, NY; Oxford Academic), 278–292. 10.1093/oso/9780190913656.003.0021

[B17] Haeyen S. (2019). Strengthening the healthy adult self in art therapy: using schema therapy as a positive psychological intervention for people diagnosed with personality disorders. Front. Psychol. 10:644. 10.3389/fpsyg.2019.00644 30967824 PMC6441831

[B18] Hu J. Zhang J. Hu L. Yu H. Xu J. (2021). Art therapy: a complementary treatment for mental disorders. Front. Psychol. 12:686005. 10.3389/fpsyg.2021.686005 34456801 PMC8397377

[B19] Hvidt N. C. Nielsen K. T. Kørup A. K. Prinds C. Hansen D. G. Viftrup D. T.. (2020). What is spiritual care? Professional perspectives on the concept of spiritual care identified through group concept mapping. BMJ Open 10:e042142. 10.1136/bmjopen-2020-042142 33372078 PMC7772306

[B20] Kaimal G. Jones J. Dieterich-Hartwell R. Wang X. (2021). Long-term art therapy clinical interventions with military service members with traumatic brain injury and post-traumatic stress: findings from a mixed methods program evaluation study. Milit. Psychol. 33, 29–40. 10.1080/08995605.2020.1842639 PMC10013461 38536339

[B21] Kirca B. (2019). Spiritual dimension in art therapy. Spirit. Psychol. Couns. 4, 257–274. 10.12738/spc.2019.4.3.071

[B22] Kruse A. Schmitt E. (2019). “Spirituality and transcendence,” in The Cambridge Handbook of Successful Aging, eds R. Fernández-Ballesteros, A. Benetos, and J.-M. Robine (Cambridge: Cambridge University Press), 426–454. 10.1017/9781316677018.025

[B23] Lalani N. (2020). Meanings and interpretations of spirituality in nursing and health. Religions 11:428. 10.3390/rel11090428

[B24] Lefèvre C. Ledoux M. Filbet M. (2016). Art therapy among palliative cancer patients: aesthetic dimensions and impacts on symptoms. Palliat. Support Care 14, 376–380. 10.1017/s1478951515001017 26584521

[B25] Lev M. (2020). Art as a mediator for intimacy: reflections of an art-based research study. J. Appl. Arts Health 11, 299–313. 10.1386/jaah_00041_7

[B26] López-Tarrida Á. C. de Diego-Cordero R. Lima-Rodríguez J. S. (2021). Spirituality in a doctor's practice: what are the issues? J. Clin. Med. 10:5612. 10.3390/jcm10235612 34884314 PMC8658590

[B27] Malchiodi C. A. (2020). Trauma and Expressive Arts Therapy: Brain, Body, and Imagination in the Healing Process. New York, NY: Guilford Press.

[B28] Martins D. (2013). Art therapy and the symbolic and creative potential of artistic mediators (Master thesis presented in Faculty of Fine Arts). University of Lisbon, Lisbon. Available online at: https://repositorio.ul.pt/handle/10451/10008 (accessed December 20, 2022).

[B29] Niemiec R. M. Russo-Netzer P. Pargament K. I. (2020). The decoding of the human spirit: a synergy of spirituality and character strengths toward wholeness. Front. Psychol. 11:2040. 10.3389/fpsyg.2020.02040 33013513 PMC7498576

[B30] Post L. Ganzevoort R. R. Verdonck-de Leeuw I. M. (2020). Transcending the suffering in cancer: impact of a spiritual life review intervention on spiritual re-evaluation, spiritual growth and psycho-spiritual wellbeing. Religions 11:142. 10.3390/rel11030142

[B31] Puchalski C. M. Vitillo R. Hull S. K. Reller N. (2014). Improving the spiritual dimension of whole person care: reaching national and international consensus. J. Palliat. Med. 17, 642–656. 10.1089/jpm.2014.9427 24842136 PMC4038982

[B32] Reed P. G. (2009). Demystifying self-transcendence for mental health nursing practice and research. Arch. Psychiatr. Nurs. 23, 397–400. 10.1016/j.apnu.2009.06.006 19766931

[B33] Roze des Ordons A. L. Sinuff T. Stelfox H. T. Kondejewski J. Sinclair S. (2018). Spiritual distress within inpatient settings - a scoping review of patients' and families' experiences. J. Pain Sympt. Manage. 56, 122–145. 10.1016/j.jpainsymman.2018.03.009 29548894

[B34] Ryff C. (2021). Spirituality and well-being: theory, science, and the nature connection. Religions 12:914. 10.3390/rel12110914 34881052 PMC8651234

[B35] Sánchez García L. Pinna-Perez A. (2021). Expressive flamenco©: an emerging expressive arts-based practice. Am. J. Dance Ther. 43, 3–35. 10.1007/s10465-020-09339-2 33500594 PMC7819147

[B36] Uttley L. Stevenson M. Scope A. Rawdin A. Sutton A. (2015). The clinical and cost effectiveness of group art therapy for people with non-psychotic mental health disorders: a systematic review and cost-effectiveness analysis. BMC Psychiatry. 15:151. 10.1186/s12888-015-0528-4 26149275 PMC4493800

[B37] Warth M. Kessler J. Hillecke T. Bardenheuer H. J. (2016). Trajectories of terminally ill patients' cardiovascular response to receptive music therapy in palliative care. J. Pain Sympt. Manage. 52, 196–204. 10.1016/j.jpainsymman.2016.01.008 27090850

[B38] Xu Y. Shao J. Zeng W. Wu X. Huang D. Zeng Y.. (2021). Depression and creativity during COVID-19: psychological resilience as a mediator and deliberate rumination as a moderator. Front. Psychol. 12:665961. 10.3389/fpsyg.2021.665961 34025527 PMC8134673

